# Identification of BRCA1 Deficiency Using Multi-Analyte Estimation of BRCA1 and Its Repressors in FFPE Tumor Samples from Patients with Triple Negative Breast Cancer

**DOI:** 10.1371/journal.pone.0153113

**Published:** 2016-04-14

**Authors:** Aruna Korlimarla, Jyothi S. Prabhu, Jose Remacle, Savitha Rajarajan, Uma Raja, Anupama C. E., B. S. Srinath, Suraj Manjunath, Gopinath K. S., Marjorrie Correa, Prasad M. S. N., T. S. Sridhar

**Affiliations:** 1 Division of Molecular Medicine, St. John’s Research Institute, Bangalore, India; 2 Sri Shankara Cancer Hospital & Research Centre, Bangalore, India; 3 Department of Surgical Oncology, St. John’s Medical College and Hospital, Bangalore, India; 4 Rangadore Memorial Hospital, Bangalore, India; 5 Department of Pathology, St John’s Medical College and Hospital, Bangalore, India; University of Torino, ITALY

## Abstract

**Purpose:**

Apart from germ-line BRCA1-mutated breast cancers, a significant proportion of women with sporadic triple negative breast cancer (TNBC) sub-type are known to harbour varying levels of BRCA1-dysfuction. There is currently no established diagnostic method to identify these patients.

**Methods:**

The analysis was performed on 183 primary breast cancer tumor specimens from our longitudinal case-series archived as formalin-fixed-paraffin-embedded (FFPE) blocks comprising 71 TNBCs and 112 Hormone receptor positive HER2 negative (HR+HER2-) tumors. Transcript levels of BRCA1 and two of its repressors ID4 and microRNA182 were determined by TaqMan quantitative PCR. BRCA1 protein was detected immunohistochemically with the MS110 antibody.

**Results:**

The representation of BRCA1 and its repressor ID4 as a ratio led to improved separation of TNBCs from HR+HER2- compared to either measure by itself. We then dichotomised the continuous distribution of each of the three measurements (Protein, MIRNA and transcript:repressor ratio) into categories of ***deficient (0)*** and ***adequate (1)***. A composite BRCA1 Deficiency Score (BDS) was computed by the addition of the score for all three measures. Samples deficient on 2 or more measures were deemed to be BRCA1 deficient; and 40% of all TNBCs met this criterion.

**Conclusion:**

We propose here a simple multi-level assay of BRCA1 deficiency using the BRCA1:ID4 ratio as a critical parameter that can be performed on FFPE samples in clinical laboratories by the estimation of only 3 bio-markers. The ease of testing will hopefully encourage adoption and clinical validation.

## Introduction

A decade separated the linking of germ-line BRCA1 mutations to familial breast and ovarian cancer [1] and the hallmark review by Turner et al, [2] wherein they suggested that somatic disruption of the function of the same genetic pathways might be encountered in a sub-set of sporadic breast cancers. They termed this phenocopy, “BRCAness”. The dysfunction of this pathway is now known to lead to ***impaired Homologous-Recombination (HR) mediated Double-Strand Break (DSB) repair***, and as a result leads to significant genomic instability (GI) in these tumors.

It has been noted that clinically Triple Negative Breast Cancers (TNBC), (tumors that lack the expression of ER, PR and do not have HER2 over-expression) and the molecular class of Basal-like breast cancers (BLBC) have a significant overlap. Phenotypically most BRCA1-mutated tumors are of the TNBC/BLBC class, in not expressing luminal markers, being more often high grade, highly proliferative and carrying mutations of TP53 [3]. The mechanisms of BRCA1 silencing in sporadic TNBCs and BLBCs are known to comprise the full spectrum of genetic and epigenetic mechanisms, including somatic mutations, transcriptional repression, microRNA based down-regulation or translational blockade, and generation of alternately spliced-variants.

The increased representation of TNBCs in certain ethnic groups such as African-Americans and populations like Indians is well documented [4–6]. However it is known that BRCA1 deficiency is present only in a sub-set of all sporadic TNBC/BLBCs [7].

Numerous technical approaches have been advocated for the identification of sporadic TNBCs that are deficient in BRCA1 function and as a consequence have defective HR mediated DSB repair. While the loss of RAD51 focus formation is a robust marker of HR deficiency in cell-lines, it has not been possible to perform this test reliably on clinical specimens in the routine lab setting.

An alternate strategy has been the identification of a molecular signature in BRCA1-mutated patients and look for a matching signature in sporadic TNBCs. Examples of this approach are 1) Vollebergh MA et al., proposed an array CGH classifier derived from BRCA1-mutated cancer patients and subsequently tested it on predicting response to platinum in HR+HER2-negative breast cancer patients [8]. 2) Konstantinopolus et al, proposed a 60-gene gene-expression profile of BRCAness that correlates with response to chemotherapy in epithelial ovarian cancers [9].

While there is obvious merit in each of the proposed tests, we wished to avoid a strategy based on resemblance to BRCA1-mutated tumors for reasons outlined later. Instead we have directly measured the repressors of BRCA1 (ID4 and MIR182) and the level of BRCA1 transcripts from the tumors. Our emphasis was on the development of a simple test that could be performed on FFPE specimens in a clinical lab that is involved in routine molecular testing.

While we acknowledge that the ideal test would be one that demonstrates functional deficiency of HR mediated DSB repair in fresh clinical specimens, we have worked towards a practical strategy that would permit the identification of tumors with deficient BRCA1.

We have developed a composite measure of BRCA1 deficiency that measures BRCA1 at multiple levels and is based on the discovery that the ratio of BRCA1:ID4 provided superior segregation of HR+HER2- from TNBCs than the levels of either transcript by themselves. The resulting BRCA1 Deficiency Score (BDS) permits the separation of tumors likely to have adequate BRCA1 from those with little or no BRCA1.

The obvious clinical implication of such a test stems from the results of the CALGB-40603 which reported a significant increase in the pathologic-complete response (pCR) rate from 44% to 60%, upon the addition of carboplatin to standard neo-adjuvant chemo therapy (NACT) in women with stage II and III TNBC [10]. It is possible that sub-selection of TNBC patients with BRCA1 deficiency could improve these response rates even further.

## Materials and Methods

### Ethics statement

The patients from whom the samples in this study were collected were subjects enrolled into a prospective observational study conducted at two tertiary cancer care hospitals in Bangalore, India. The research study was approved by the respective institutional review boards of our collection centers—St John’s National Academy of Health Sciences Institutional Ethics Committee (IRB protocol # 62/2008) and Rangadore Memorial Hospital Ethics Committee (IRB protocol # RMHEC/02/2010). All patients signed a written informed prior to participation. The consent procedure was also approved by the institutional ethics review boards from the tertiary cancer care hospitals where the subjects were enrolled.

### Breast cancer cohort and specimens used for molecular analysis

The overall cohort has 446 patients who were recruited at first diagnosis over a 5 year period, between June of 2008 and February of 2013. These patients are being actively followed up by a dedicated breast cancer support group “Aadhara”, through which we have achieved an extraordinary follow-up rate of 97% over the past 7.5 years, and the median follow-up of the cohort is 59 months as of November 30^th^ 2015. Tumor tissue samples were collected at the time of surgery and fixed in buffered neutral formalin and processed as paraffin embedded blocks. Sections were cut from these blocks, stained with haematoxylin and eosin and examined by a pathologist to confirm the presence of tumor. Immunohistochemistry for ER, PR and HER2 were done for determining receptor status by standard procedures. Tumors which had higher than 10% of cells stained for ER and PR were considered positive.

All tumors which were HER2 positive and HER2 equivocal by IHC were tested by FISH for HER2 according to standard procedures. Only tumors that had ratio of more than 2.2 were considered amplified and HER2 positive [11].

**Fig 1:**
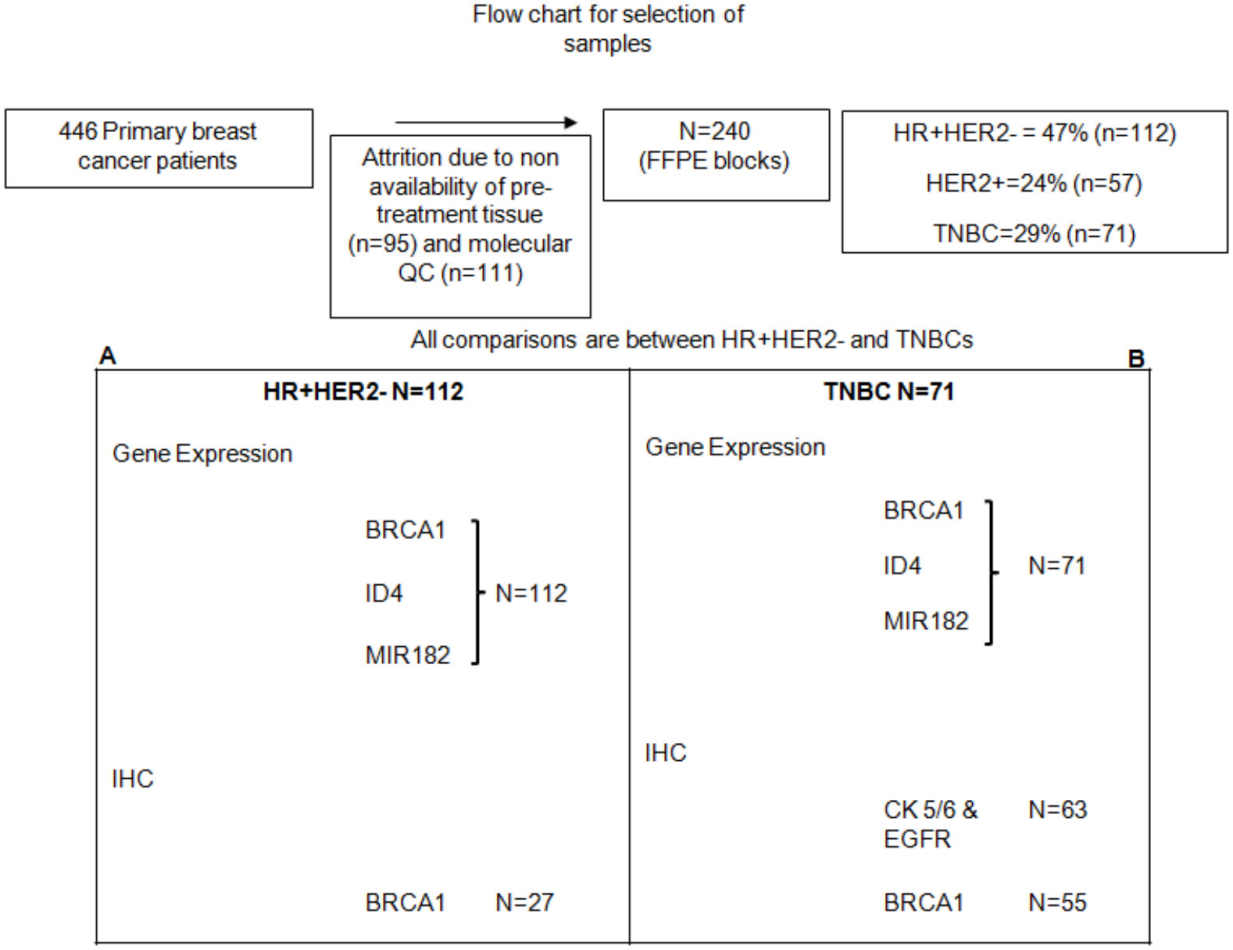
Identification of BRCA1 deficiency using multi-analyte estimation of BRCA1 and its repressors in FFPE tumor samples from Indian patients with Triple Negative Breast Cancer. Flow chart depicting sequential elimination of tumors based on availability and QC criteria and the proportions of the sub-types used in this study. Panel A & B show the detailed break-up of numbers from each of the sub-types used in the different analytical tests. HR+: Hormone Receptor positive, TNBC: Triple Negative, QC: Quality Control.

Only FFPE blocks containing pre-treatment tissue with greater than 50% cancer epithelial cells were used for molecular analysis. Selection of samples for each molecular measurement is outlined in Fig 1. All clinical and histopathological details have been collected from the medical-records and updated at regular intervals. Radiologically recorded distant metastases or histologically confirmed local recurrence and death related to disease have been documented.

### Extraction of RNA and reverse transcription of RNA and microRNA

The methods used for nucleic acid extraction and quantitative Real time-PCR, selection of housekeeping genes (HKG) and quality control criteria for inclusion of samples in this analysis have been described in detail in a recent publication from our lab [12]. In brief, total ribonucleic acid (RNA) was extracted from two 20μm sections from each patient’s tumor block and the sections were deparaffinised by heat, and then subjected to overnight digestion using proteinase K (Qiagen #19133). Total RNA was then extracted using Tri Reagent protocol according to manufacturer instructions (Sigma Aldrich # T9424). Quantitation of the RNA was done using the Ribogreen dye (Invitrogen # R11490- Quant –iT Ribogreen RNA assay kit) on a fluorescent plate reader (Tecan M200-Pro Infinite Series). 500ng of total RNA was then reverse transcribed using the ABI high capacity cDNA archive kit (ABI # 4322171) as per the manufacturer’s protocol.

MicroRNA present in total RNA extracted as given above was converted to cDNA using stem-loop primers specific for the chosen microRNA according to published protocols, The TaqMan microRNA Reverse Transcription Kit (Applied Bio systems, #4366596) was used for cDNA conversion. Concentration of 50ng/μl of total RNA was used for the conversion of microRNA to cDNA according to manufacturer's instructions using Verity 96 well thermal cycler (Applied Biosystems). Briefly, the reverse transcription reaction mixture was incubated at 16°C for 30 minutes, 42°C for 30 minutes, 85°C for 5 minutes and finally held at 4°C.

### Gene expression (mRNA and microRNA) by qPCR and normalization

The expression level of test genes was determined along with a panel of 3 reference genes (*PUM1*, *RPL13A*, *ACTB*). Using 5ng cDNA template per reaction real time PCR was done in duplicate using TaqMan qPCR chemistry on the Light Cycler 480 II (Roche Diagnostics). Total RNA Universal Human Reference RNA (Agilent, # 740000) was also reverse transcribed and 1ng of this template was run in the assay as a control. 5 ng cDNA from each sample is included in a total reaction volume of 10μl. Pre-incubation and initial denaturation of the template cDNA was performed at 95°C for 10 min, followed by amplification for 45 cycles at 95°C for 15 sec and 60°C for 1 min. All samples which had average Ct value of three housekeeping genes beyond 2 SDs above the mean were excluded from further analysis, as these specimens have very poorly preserved RNA.

TaqMan microRNA inventoried assays for qRT-PCR (Applied Biosystems, #4427975) were used for each of the test and control MIRNA (RU48 and the test microRNA—MIR182). These assay kits comprise stem loop primers for cDNA conversion as well as TaqMan primer-probes for RT PCR analysis. 50 ng of total RNA was reverse transcribed for using control as well as test stem loop primers of a control microRNA, and then 2.5ng of each microRNA-cDNA is included in a total PCR reaction mixture of 10μl. Each microRNA analysis by qRT PCR was subjected to an enzyme activation step at 95°C for 10 minutes, followed by 45 cycles of denaturation (95°C for 15 seconds) and annealing (60°C for 60 seconds). The assay IDs for each of the tested assay for gene expression and microRNAs are given in Table 1.

**Table 1:** Assay part numbers (Gene expression and MicroRNA analysis).

Assay Id	Part number
BRCA1	Hs00173233_m1
ID4	Hs02912975_g1
ACTB	Hs00357333_g1
RPL13A	Hs03043885_g1
PUM1	Hs00472881_m1
MIR182	hsa-miR-182- 002334
RNU-48	RNU48- 001006

240 specimens (112 HR+HER2-ve, 57 HER2+ve and 71 TNBCs) fulfilled all of the QC criteria including greater than 50% of cells being tumor epithelial cells, adequate RNA for reverse transcription and average housekeeping gene values below the cut-off indicating adequate preservation of transcripts in the samples. Breast cancers that were clinically HER2 positive were excluded for this analysis. Therefore, the results presented are for 183 samples (112 HR+HER2-ve and 71 TNBCs). The clinical characteristics of this sample set are provided in Table 2.

**Table 2:** Clinical characteristics.

Clinical characteristics	HR+HER2- N = 112 (%)	TNBC N = 71 (%)
% IDC	93	93
Median age (yrs)	60	54
Median tumor size (cm)	3	3.5
LN Positive	73 (65)	35 (49)
LN Negative	33 (29)	34 (48)
Grade I	14 (12)	3 (4)
Grade II	53 (47)	25 (35)
Grade III	39 (36)	39 (55)
Family history <60y	5	2

To determine relative transcript abundance, the Ct values for the test gene were normalized to the mean Ct value of the three reference genes for each sample as ΔCt. The Relative Normalized Units (RNU) of expression of the test genes was calculated as 15- ΔCt. From each of the test genes in the study, we took the lowest value (x) of the 183 specimens, subtracted 1 from it and subtracted that value (x-1) from all the RNU values. This allowed us to obtain a series starting from 1 with no negative value for all transcript measurements. This value is referred to as aRNU (adjusted Relative Normalised Units). The dynamic range for transcript measurements was about 2^12^ (4000 fold).

### Immunohistochemistry

Immunohistochemistry was done for BRCA1 according to standard procedures. Briefly, sections (5μ in thickness) were cut from FFPE blocks on poly L-lysine coated slides and subjected to deparaffinization in xylene and rehydrated in graded alcohol. After blocking endogenous peroxidase with a 3% hydrogen peroxide solution, antigen retrieval was done in 0.01M EDTA buffer at pH 8, in a microwave at 800 W for 2 min, 480 W for 7 min followed by 160 W for 11 min. Primary blocking was done with 3% bovine serum albumin (BSA, Sigma) for 30 min at room temperature. Primary antibody for BRCA1 (Clone MS110, Calbiochem Cat# OP92) at 1:100 dilution was applied for 1 hr at room temperature. Sections were further incubated with secondary antibody (DAKO REAL^™^ EnVision ^™^) for 20 min as per the kit instructions, followed by development of the colour using DAB (DAKO REALTM EnVision TM) for 10 min. Sections were counterstained with hematoxylin and mounted after dehydration in graded alcohol and xylene. Appropriate positive and negative controls were run for each batch.

After determining receptor status, all TNBC samples were tested for CK5/6 and EGFR to further divide them into Basal and non-Basal groups. The procedure for IHC was exactly like the one used for BRCA1 IHC. The antibodies used were Cytokeratin 5/6 (Clone D5/16 B4, DAKO Cat #-IS780) and EGFR (Clone 31G7, Invitrogen, Cat # 08 1205). For Cytokeratin 5/6, complete cytoplasmic and membrane staining in more than 10% of the cancer cells was taken as positive. Membranous staining of >1% of the epithelial cells was considered as positive for EGFR. IHC images of Cytokeratin 5/6 and EGFR are presented in S2 Fig.

### Statistical Analysis

The normal distribution of continuous data was examined using Q-Q plots and histograms. Students t test was used to examine the relation between the gene markers tested when data was normally distributed. When not normally distributed Mann Whitney U test and Spearman’s rank correlation were used. *P* values less than 0.05 was considered statistically significant. All analyses were performed using XLStat 2014.

## Results

As one of the three most intensely scrutinized genes in breast cancer (the other two being *ESR1* and *HER2*), there is no molecular mechanism of *BRCA1* regulation that has not been examined in primary human breast cancer specimens. These molecular regulatory mechanisms include, 1) promoter hyper-methylation, 2) transcriptional repression, 3) alternative-splicing, 4) MIRNA based regulation and 5) protein localization. We have analyzed all regulatory mechanisms except for alternative splicing. However, in this report we present the results of only transcriptional repression, MIR regulation and protein localization, and not that of promoter methylation, since virtually all the methylated specimens had demonstrable deficiency of BRCA1 transcripts and/or protein.

### Gene expression of *BRCA1*

**Fig 2:**
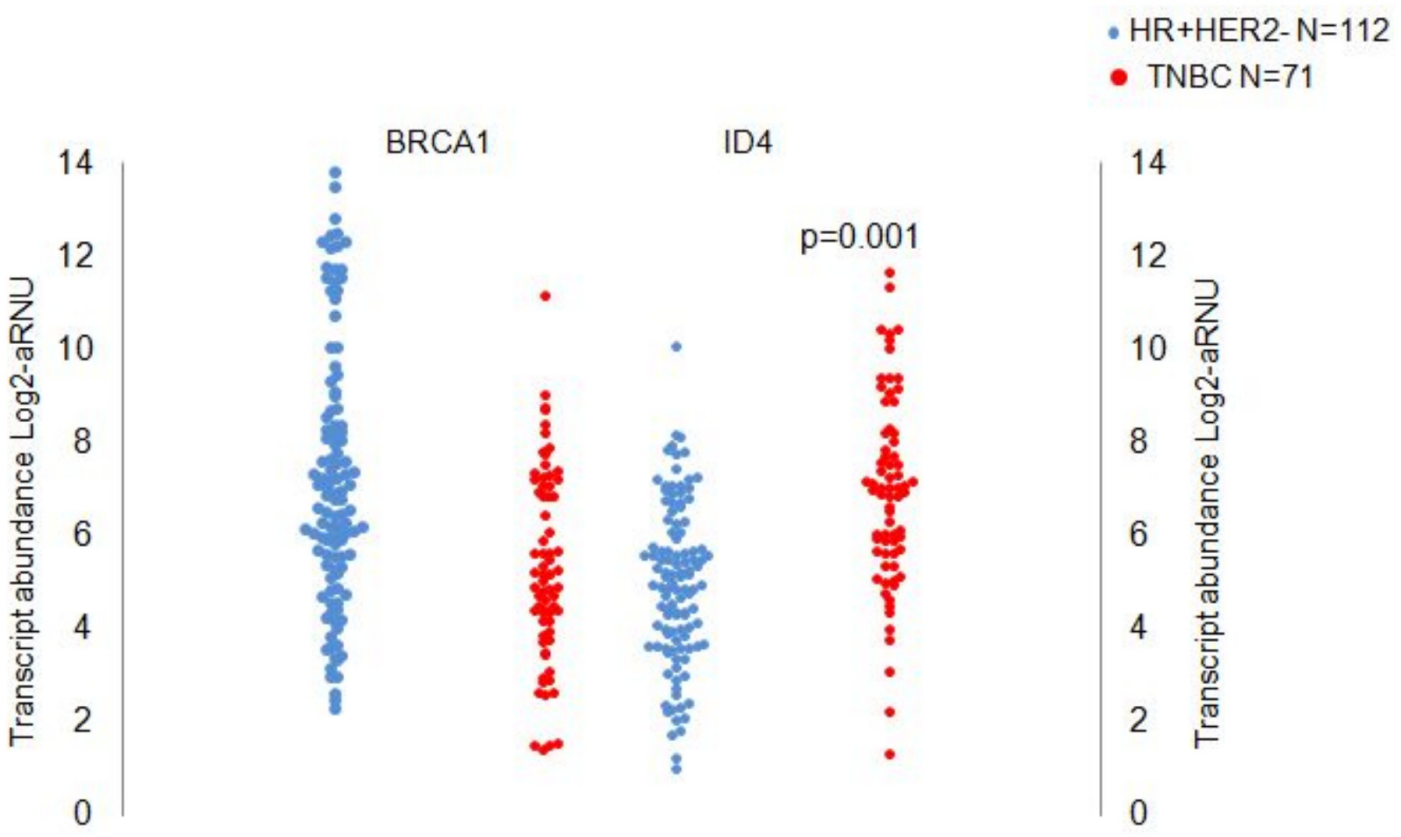
Transnscript abundance of BRCA1 and ID4 in TNBC Vs HR+HER2-ve tumors. Transcript abundance of BRCA1 and ID4 in HR+HER2-veand TNBCs. Median expression of BRCA1 and ID4 transcripts is significantly different in the two groups; *p = 0.008 for BRCA1, and **p = 0.001 for ID4, (Mann Whitney U Test). The high ranges of BRCA1 are exclusively HR+, and the high-ranges of ID4 are all TNBCs.

As with all genes there is a considerable variation in the abundance of BRCA1 transcript across all specimens, with a range of 12 log2 units or approximately 4000 fold. BRCA1 distribution across the specimens was not “normally-distributed” as is the case with most transcripts, but exhibited a characteristic bimodal pattern. (S1A Fig) On further scrutiny it turned out that except for a single TNBC specimen, the top third of the distribution (approximately 4 Cts) was exclusively comprised of HR+ve specimens. This group comprised approximately a quarter of all HR+ve tumors. The mean expression of BRCA1 in the HR+ve group was approximately four-fold higher compared to TNBCs (7.17 *Vs* 5.38 log2 units). As seen in the scattergram (Fig 2), the pattern of expression is significantly different between the groups. (p = 0.008). However, the significant overlap in transcript abundance between the two groups in the lower half of the range indicated that a small but significant fraction of HR+ve tumors had low BRCA1 transcript abundance. Biologically this suggests that these luminal tumors manage to function with low levels of BRCA1 transcripts.

Therefore, while high BRCA1 transcript levels are a reliable measure of adequate BRCA1, the use of BRCA1 transcript levels in and of itself is not able to discriminate samples with low but adequate levels from those that are low and deficient. As expected a small but significant number of TNBCs have BRCA1 transcript abundance values beyond even the mean value of HR+ tumors, suggesting that at least some TNBCs have adequate BRCA1.

### Transcriptional repression of BRCA1 by ID4

More than a decade ago it was experimentally demonstrated in a human ovarian cancer cell line system that ID4 was a potent negative regulator of BRCA1 [13]. ID proteins are known to be key regulatory elements acting through negative regulation of gene transcription to block cell differentiation. ID4 is a dominant-negative basic helix-loop-helix (bHLH) protein which has only the dimerization motif but lacks the DNA binding domain, and helps antagonize the action of other bHLH transcriptional factors by forming transcription incompetent heterodimers [14]. More recently Brogie’s group have shown that in a large series of primary breast cancers at the Memorial Sloan Kettering Cancer Center (MSKCC) 40% of 101 TNBCs had high levels of ID4 immunoreactivity compared to only 5% of 113 HR+ve tumors [15]. In order to examine the contribution of ID4 to the BRCA1 status in our series of tumors we measured ID4 transcripts in these tumors.

We observed a flipped distribution of ID4 transcript compared to BRCA1 abundance in the two tumor classes (Fig 2). High ID4 expression correlated with TNBC group (p = 0.001). The upper third of the range of ID4 transcript values was comprised almost exclusively of TNBCs. However, the middle third of the distribution had significant numbers of tumors of both classes and the lower 4 log2 units of the distribution comprised almost exclusively HR+ve tumors. (S1B Fig) shows their distributions and clear reciprocal relationship.)

### Relationship between BRCA1 and ID4 transcript levels

**Fig 3:**
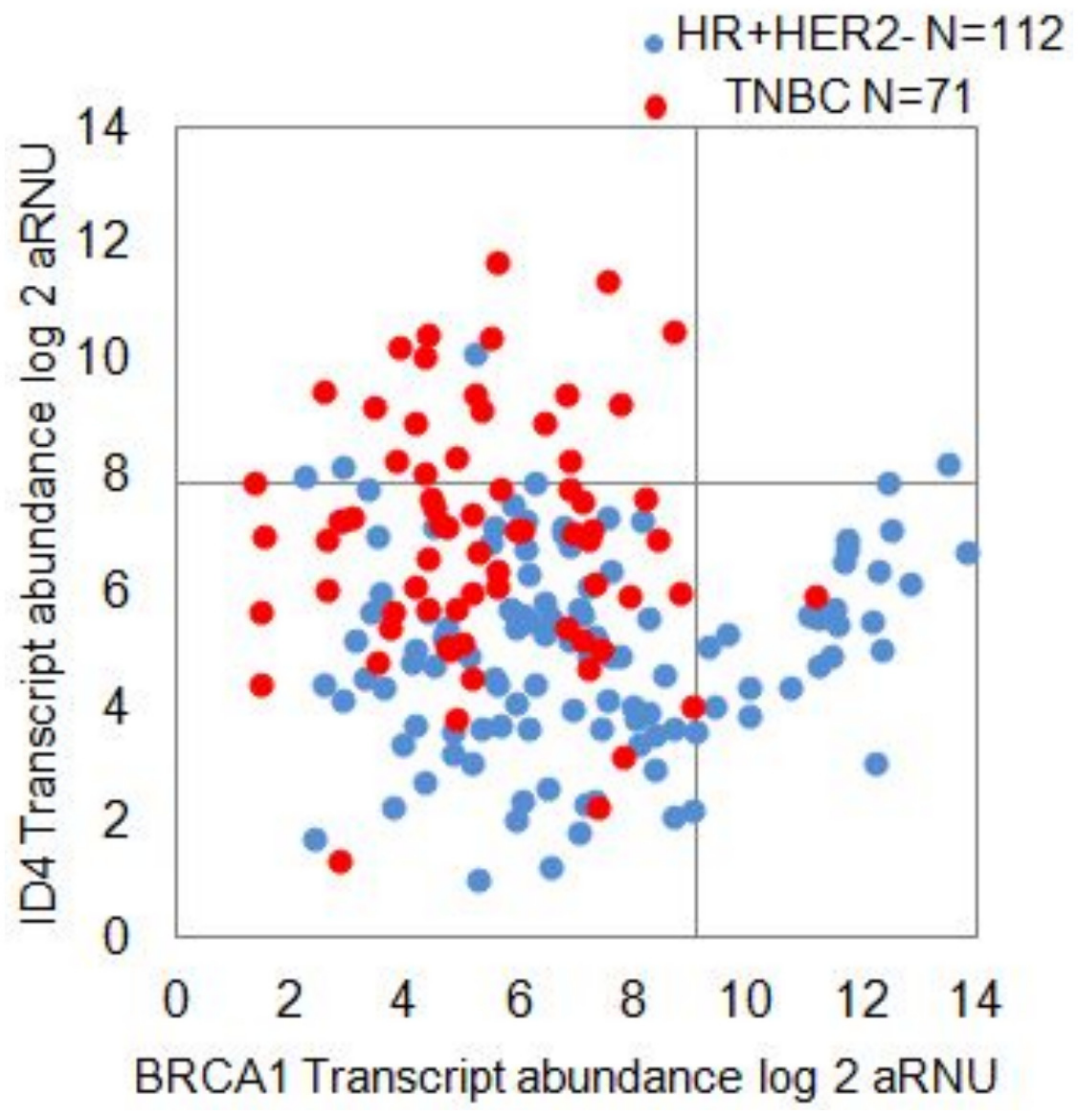
Relationship between BRCA1 and ID4. Relationship between BRCA1 and ID4.A four quadrant plot with “cut-off” lines for both transcripts that are at 1 SD above the mean value of all specimens. The upper right quadrant representing high ID4 and high BRCA1 is empty. The upper left quadrant of high ID4 and low BRCA1 is almost exclusively TNBCs while the lower right quadrant of high BRCA1 and low ID4 is exclusively luminal tumors.

To examine the relationship between these two transcripts in individual tumor specimens, we plotted the transcript abundances of BRCA1 versus ID4 (Fig 3). There was no obvious direct or inverse relationship between the transcript levels of the two genes. We then defined for each gene, values 1 SD above the mean, as a cut-off, beyond which specimens were considered to have “high-levels” of the transcript. The cut-off values established this way were 9.1 (6.4 + 2.6) for BRCA1 and 7.8 (5.73 + 2.08) for ID4. When the scatterplot was represented with these cut-offs, a four quadrant plot was generated. The upper right quadrant representing high BRCA1 and high ID4 was virtually empty indicating that a high-ID4 expression was not compatible with high expression of BRCA1. In contrast the lower right quadrant representative of high levels of BRCA1 and low ID4 was almost entirely filled with HR+ve tumors. Conversely the upper left quadrant representing high ID4 transcript levels and low BRCA1 transcripts was filled with mostly TNBCs. The lower-left quadrant representing the dual-low BRCA1 and ID4 transcripts was a mixed group of HR+ve and TNBCs.

### A novel measure to identify BRCA1 deficiency in a sub-group of samples with middle-of-the range values of both BRCA1 and ID4

Using the measurements of BRCA1 transcript levels and ID4 transcripts we were able to identify the high BRCA1 transcript containing tumors as most likely to possess adequate BRCA1 activity, and the high ID4 tumors as most likely to be BRCA1- deficient. However, these measures did not help classify the tumors with low levels of both BRCA1 and ID4.

There has been experimental demonstration, albeit *in-vitro*, that BRCA1 and ID4 have a reciprocal regulation [16], with each suppressing the expression of the other. This observation provided the basis for our idea of not using either measure alone but to use a ratio of their abundances to examine their interaction in a particular tumor.

**Fig 4:**
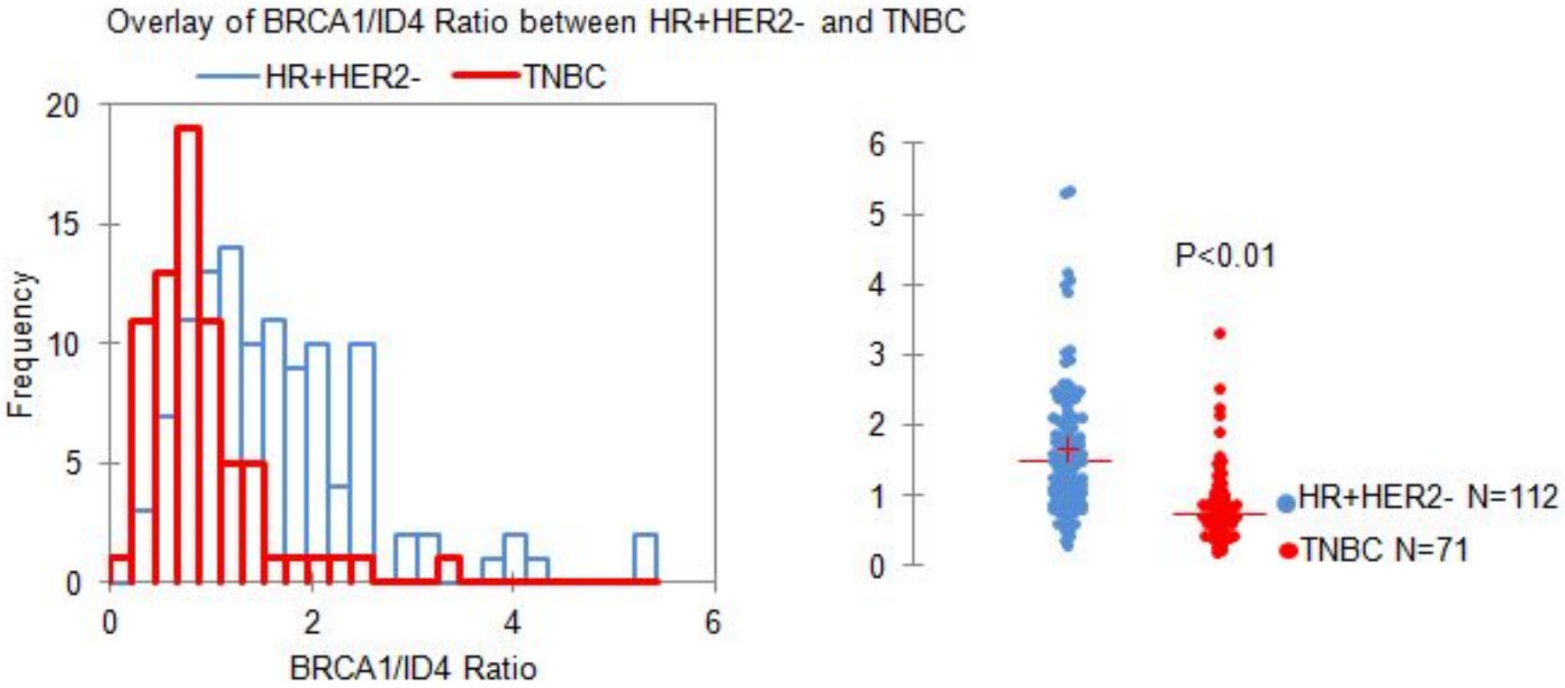
Distribution of BRCA1/ID4 ratio values in luminal tumors compared to TNBCs. Distribution of BRCA1/ID4 ratio values in luminal tumors compared to TNBCs. An overlay of the histograms in panel A clearly shows the marked “Left-Skew” of TNBCs and a relatively “Right-shifted” distribution of luminal (HR+) tumors. The median value is significantly different in the two classes (*p<0.01- Mann Whitney U test).

As expected, the ratio of BRCA1:ID4 was significantly different between HR+HER2- and TNBCs as shown in Fig 4 (p<0.01). The median ratio of the HR+HER2- class was beyond 1.5 while that of the TNBCs was half of that value at 0.74, and only a handful of TNBCs had a ratio greater than 2. When we looked at all specimens with ratios ≤0.74, the group comprised as expected of half of all TNBCs while it had only 10% of all HR+ve specimens, leading to a significant enrichment for TNBCs.

### Repression of BRCA1 by MIR182

Recently Moskwa et al., have shown that MIR182 selectively down regulates BRCA1 expression in cell lines leading to defective homologous recombination (HR) and non-homologous-end-joining (NHEJ) and renders cells hypersensitive to ionising radiation. They also showed that BRCA1 transcripts are selectively enriched in the Argonaute/MIR182 complex and antagonizing MIR182, enhanced BRCA1 protein levels and protected cells from IR-induced cell death [17].

**Fig 5:**
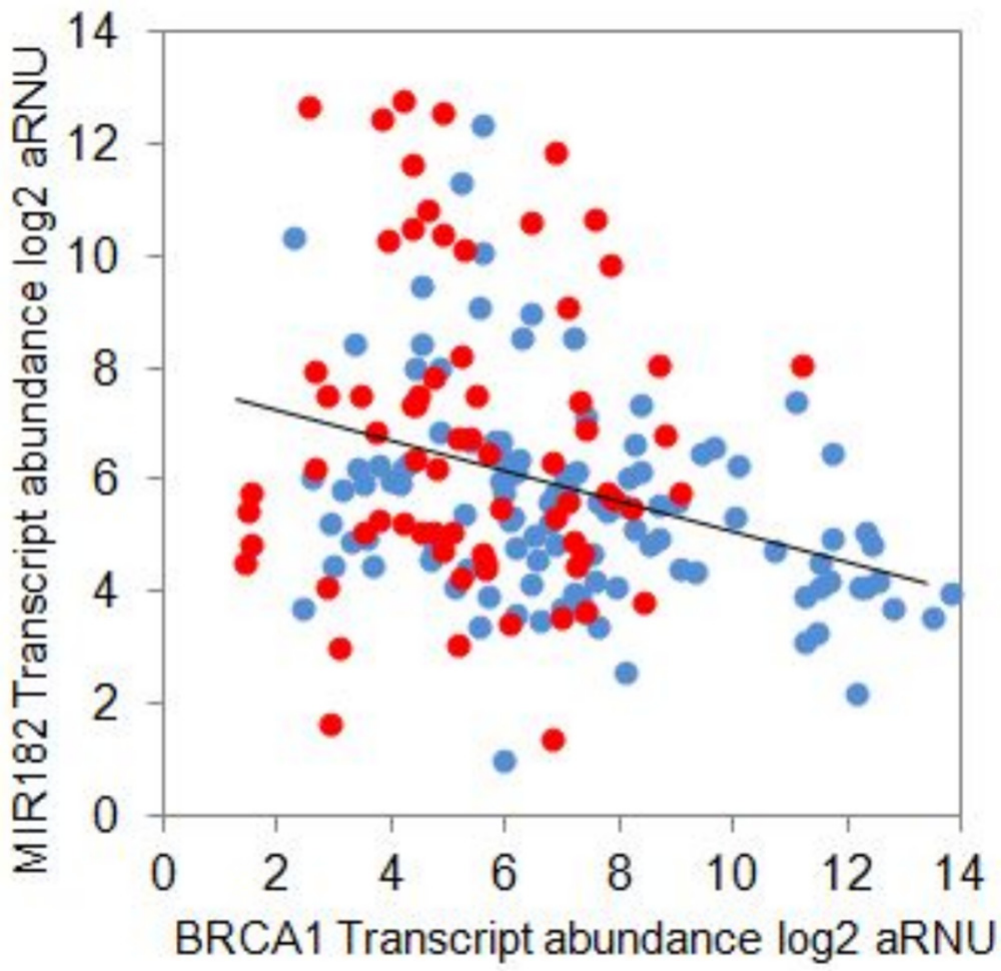
Scatter plot of expression of MIR182 *v* BRCA1:MIR182. Scatter plot of expression of MIR182 *v* BRCA1:MIR182 expression correlated to being highest in TNBC class (Pearson’s CC- −0.27, *p<0.05) There is also an inverse correlation between MIR182 and BRCA1transcripts.

On examination of the distribution of MIR182 in our specimens, we observed an inverse relationship between the abundance of BRCA1 and MIR182 transcripts and an enrichment of high MIR182 transcript in TNBCs, as shown in Fig 5 (p<0.05). We again defined a cut-off value using mean+1SD (6.03+2.28 = 8.32) for low versus high MIR182. MIR182 also showed a clear inverse relationship with the presence of BRCA1 protein indicating that it is a potential translational block as well (S3 Fig).

### BRCA1 protein detection

One of the major stumbling blocks to the use of a simple IHC assessment of BRCA1 protein as a measure of BRCA1 function has been the significant challenge in the standardization of a robust assay using a reagent with high sensitivity and specificity. Recently Milner et al., screened a panel of commercial antibodies as well as a very large panel of internally generated antibodies targeting BRCA1and found that MS110 (a mouse monoclonal antibody designed against the 304aa of the N-terminus) was best at delivering data with the expected staining profile and had the highest signal intensity for BRCA1 detection in FFPE samples [18].

Only 55 of the 71 TNBCs used for gene expression had adequate tissue available for IHCs analysis. For the positive-control, we selected 27 highly HR+ve tumors that had an average ER Allred score of 7, from mostly post-menopausal women (23/27 post-menopausal; median age = 65).

Nuclear staining of normal breast epithelial cells on the tumour sections and/or normal breast tissue sections from the same breast was used as control. BRCA1 expression in the normal epithelial cells in the samples was detected as a strong, dark brown, dotted nuclear stain (IHC images shown in (S2A Fig). Both nuclear and cytoplasmic staining pattern was observed in the tumors, matching the observations of other groups [19]. To begin with we classified the protein expression into four different classes as shown in (S2B Fig), where we considered the cytoplasmic as well as nuclear localization. However, for the purpose of this study, positive expression of BRCA1 was identified by positive staining of the nucleus in 10% or more of the neoplastic cells as outlined by Rakha et al, [19] and we disregarded the intensity of staining as it led to inter and intra observer variability. Greater than 90% (25/27) of our HR+HER2- tumors were positive for BRCA1 protein in contrast to only about half (31/55) of TNBCs.

### BRCA1 deficiency score by integration of multiple parameters

What was revealed by the measures of BRCA1 and its repressors, was that as expected TNBC tumors occupied a continuum with one extreme represented by tumors that had adequate or “high” levels of BRCA1 transcript and protein with a correspondingly low level of repressors, and the opposite end of the spectrum was occupied by tumors with complete absence of transcript and protein and a dual repression. A majority of the tumors showed varying grades of repression and a range of BRCA1 transcript and protein abundance. Therefore, we reasoned that integrating all parameters and arriving at a “BRCA1 Deficiency Score (BDS)” might be a more robust predictor of BRCA1 deficiency.

Though all of the variables measured were distributed continuously, in keeping with the general approach used in clinical-laboratory practice, we chose to dichotomise the variables into adequate and deficient classes. In the case of BRCA1:ID4 ratio and BRCA1 IHC when the value of the variable was below the cut-off and hence deemed deficient the sample in question was given a score of zero “0”, and conversely when the value of the variable was above the cut-off it was deemed adequate and given a score of one “1”. Since MIR182 has a negative effect on BRCA1 function, the reverse scaling was used with high values being deemed deficient and scored “0”.

**Fig 6:**
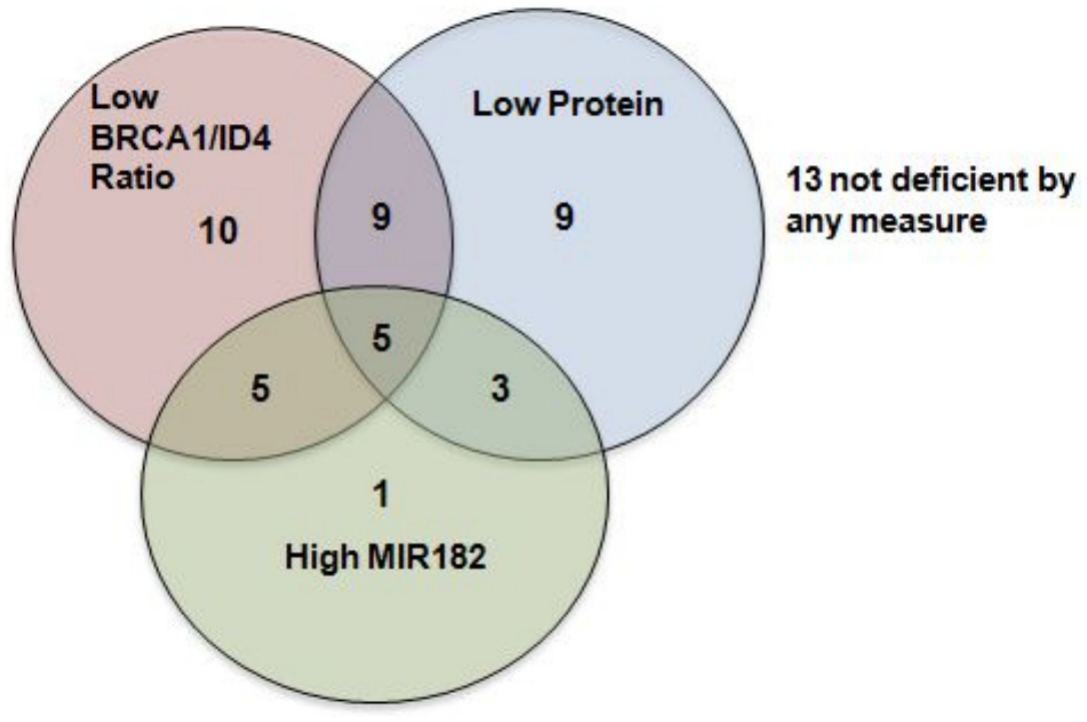
Shared BRCA1 deficiency measures in TNBCs n = 55. Shared BRCA1 deficiency measures in TNBCs n = 55.A Venn diagram, showing the overlap of TNBCspecimens deficient in BRCA1 by the three different measures.

As seen in Table 3, of the 55 TNBCs, low BRCA1/ID4 ratio and absence of BRCA1 protein was seen in approximately half of all tumors, while MIR182 seemed less of a factor and was encountered in only a quarter of all TNBCs. Not a single specimen of the 27 highly luminal tumors was deemed BRCA1 deficient by the ratio, while only a tenth had high levels of the repressor, MIR182. The cross-distribution of the TNBCs across the three variables is depicted in the Venn diagram Fig 6. Only 10% of all TNBCs were BRCA1 deficient by all measures, and in contrast a quarter of all TNBCs [20] had adequate BRCA1 function by all three measures.

**Table 3:** Distribution of TNBCs and HR+HER2- tumors adequate and deficient in the three measurements.

	Deficient Score 0 (%)	Adequate Score 1 (%)
**BRCA1:ID4 Ratio**		
Adequate: ≥0.75; Deficient: ≤0.74		
HR+HER2-	0 (0)	27 (100)
TNBC	29 (53)	26 (47)
**MIR182 aRNU**		
Adequate: ≤ 8.31; Deficient: ≥8.32		
HR+HER2-	3 (11)	24 (89)
TNBC	14 (25)	41 (75)
**BRCA1 IHC; % cells stained**		
Adequate: ≥10%; Deficient: <10%		
HR+HER2-	2 (7)	25 (93)
TNBC	26(48)	29 (52)

The percentage of tumors in each category is provided in parenthesis.

**Fig 7:**
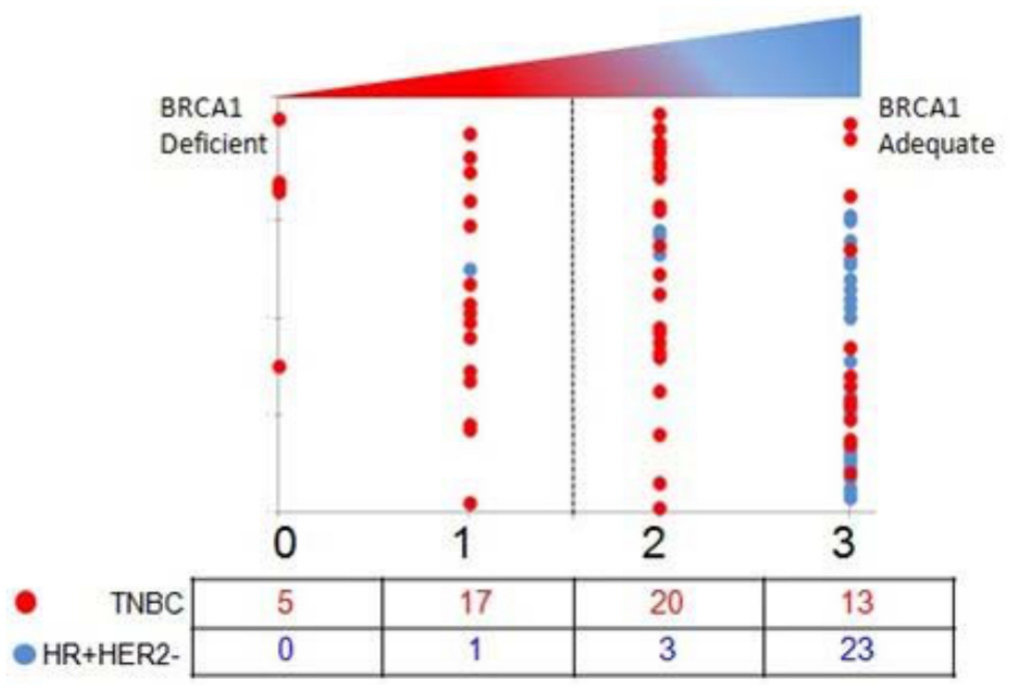
BRCA1 Deficiency Score (BDS). BRCA1 Deficiency Score (BDS): Amalgamated scores range from a low of 0 to a high of 3. Scores of 0 and 1 are almost exclusively seen in TNBCs. Scores of 2 and 3 were seen in both HR+HER2- as well as TNBCs. 23/27 HR+HER2- tumors scored adequate on all measures and hence had a score of 3. This method of selection classified 40% of TNBCs being BRCA1 deficient and only 3% of HR+HER2-ve to be BRCA1 deficient.

We then computed a composite BRCA1 Deficiency Score (BDS) by adding the individual scores for the three variables for each tumor. So the BDS would range between zero “0” on the one extreme, which would indicate absolute BRCA1 deficiency since the tumor was categorized to be deficient in all three measures, and a maximum BDS of “3” which indicated no repression and complete adequacy in all measures of BRCA1. As seen in Fig 7, five TNBCs had a BDS of 0 suggesting deficiency in all 3 measures and a further 17 had a BDS of 1 suggesting deficiency in at least 2 measures. These 22 specimens were classified as being BRCA1 deficient. In contrast none of the HR+ve tumors scored deficient on all 3 measures and a full 85% (23/27) scored adequate on all three measures.

Cheang et al in 2008 have reported 62% of their TNBCs being Basal by their 5 marker IHC (ER, PR, HER2, CK5/6 and EGFR). Their series included greater than 4000 invasive breast cancers [21]. In a more recent paper Gazinska P., et al, [22] used the terms Path-Basal, Core-Basal, and PAM50-Basal-like, to delineate TNBCs that were termed Basal by histopath, 5 IHC (including CK5/6 and EGFR) and gene-expression respectively. To rule out the possibility that the use of the 5 IHC based Core-Basal would help identify sporadic TNBCs with BRCA1 deficiency we examined the distribution of this phenotype in all of the TNBCs compared to the 22 tumors deemed to be BRCA1 deficient. The proportion slightly changes going from 58% (32/55) to 68% (15/22) of TNBCs with BRCA1 deficiency. However this would not let us substitute the Core-Basal measure for the present amalgamated “BRCA1 Deficiency Score (BDS)”.

On examination of the clinico-pathological characters of the 22 TNBC tumors deemed BRCA1-deficient compared to the set of 33 TNBCs with adequate BRCA1 a few trends emerged (S1 Table). The median age of women with BRCA1 deficiency was at 49 years, a further 5 years lesser (p<0.05) than the median age of all TNBC women (54y) which itself was 5 years under the median age of women with luminal tumors (60y). A slightly larger proportion were of grade III (63% *v* 55%) and they were marginally enriched for the Core-Basal (by IHC) class (68% *v* 51%). All of these enrichments are consistent with the trends of these variables in BRCA1 mutated tumors.

Survival analysis of the BRCA1 adequate and deficient groups did show an interesting trend with the adequate tumors showing a marginal trend towards better prognosis although this was not significant statistically (S4A Fig) due to limited numbers. Survival analysis of patients separated by IHC based classes of HR+, HER2+ and TNBC reveal the expected trends with HR+ having the best prognosis and the TNBC sub-group having the overall lowest survival. (S4B Fig).

## Discussion

The excitement upon recognizing the resemblance between familial BRCA1 mutated breast cancers and certain sporadic TNBCs (BRCAness) led to the hope that similar treatment strategies might work in both sets of patients. The early demonstration that inhibition of PARP in cells with BRCA1 mutation led to cell death [23] prompted the testing of this class of drugs in clinical trials of women with TNBCs, with discouraging results.

Right from the time of the coining of the TNBC category, it has been recognized that as a clinical group there is significant heterogeneity [24]), but most trials did not include an appropriate molecular test to sub-stratify the patients.

The obvious first approach would be to replace the IHC based TNBC classification with the molecularly defined BLBC classification; and believe that since it is a homogeneous class at the level of gene-expression, all BLBCs are BRCA1 deficient. However, in their recent elegant analysis of all of the BLBCs in the TCGA, Prat A., et al, [25] demonstrate that even after excluding both germ-line and somatic BRCA1-mutated tumors and considering only wild-type BRCA1 containing BLBCs, they fall into two distinct classes based only on the basis of BRCA1 transcript levels. Even the use of the 5-IHC based “core-basal” is not able to pick only tumors with low BRCA1 levels.

The currently known BRCAness tests (aCGH and the 60-gene expression based) were both developed based on the detection of patterns in sporadic tumors that are signatures of BRCA1 mutated tumor [9, 20]. Lips EH et al have shown that germline mutation of BRCA1 and BRCA1 hypermethylation are mutually exclusive and so we reasoned that the use of BRCA1 mutated tumors as guides for the detection of other regulatory mechanisms was likely to lead to erroneous conclusions [20].

Instead, our strategy to identify BRCA1 deficiency was based on the following three observations and assumptions. 1) A majority of BRCA1 mutated tumors are of the ER negative phenotype [26]. 2) A recent report of a massive analysis of more than 50,000 breast cancers stated that the refined histopathological predictor for BRCA1 mutation with the highest likelihood-ratio (LR; 3.43–4.41) is the TNBC phenotype [27]. 3) A significant proportion of TNBCs ***do not*** have BRCAness [20]. Therefore, we reasoned that parsing of breast cancers based on BRCA1 related measurements that led to the ***exclusion of most HR+ tumors and inclusion of only part of the TNBCs*** were likely to enrich for BRCA1 deficiency.

We reasoned that a straight forward approach to measurement of BRCA1 itself might be better. The availability of a sensitive and specific antibody with appropriate cut-offs has been a time tested approach to the stratification of clinical FFPE specimens into positive and negative groups, for eg. ER. However, the challenges of the MS110 clone are too well documented for it to be used as a single analyte for the estimation of BRCA1 levels [18].

Q-RT-PCR based measurements especially with TaqMan chemistry is the gold standard for estimation of transcript abundances. The linearity and high dynamic range of this method are additional advantages. Attempts at using low transcript abundance as indicative of BRCA1 deficiency were not feasible for a significant number of HR+HER2- tumors possessed very low BRCA1 transcript levels. It was clear that these tumors had no features of BRCA1 deficiency at the level of pathological features (grade) or in their ultimate clinical outcomes as shown in our Kaplan-Meier survival function based on IHC sub-types (S4B Fig).

The real advance in this study is the discovery that upon using the ratio of the fate determinant ID4 and BRCA1 within a tumor as a derived measure, we were able to discriminate HR+HER2- tumors with low BRCA1 and no obvious phenotypic consequences, from those tumors in which the low BRCA1 was likely to be seen in the context of a high-grade, TNBC tumor, in a younger woman; all characteristics that are strongly associated with BRCA1 dysfunction. As stated in the results section ID4 is a dominant-negative basic helix-loop-helix (bHLH) protein which has only the dimerization motif but lacks the DNA binding domain, and helps antagonize the action of other bHLH transcriptional factors by forming transcription incompetent heterodimers [14]. The truly innovative aspect of the present test is not the selection of the markers per se, which have been established as useful by multiple studies [3], but a novel method of determining BRCA1 transcript adequacy.

There is a large body of data that BRCA1 deficiency leads to the Basal-like phenotype and is rarely seen in strongly ER+ tumors [2,7,19]. In fact, Junankar et al have identified a mechanistic basis, where they show that ID4 is a key regulator of cell fate and a determinant for the cell to acquire a basal state by suppressing factors required by the cell for luminal differentiation. We speculate that this could be the reason for the BRCA1:ID4 ratio to be a better discriminator in our analysis rather than the transcript levels separately [28]. Secondly, it has been repeatedly shown that sporadic TNBC/BLBC are comprised of both BRCA1 adequate and deficient sub-groups [29]. Hence, the criteria we have adopted for evaluation of all three measures as being able to segregate BRCA1 adequacy from deficiency are i) its ability to exclude almost all luminal tumors and ii) segregate TNBCs into groups with and without adequate BRCA1.

Further we developed a composite measure of BRCA1 dysfunction, the “BRCA1 Deficiency Score (BDS)” by the amalgamation of the three parameters, the BRCA1:ID4 ratio, MIR182 levels, and BRCA1 protein presence. The continuous range of values for each parameter was then dichotomised into “adequate” and “deficient” ranges. Specimens that scored deficient in at least two out of the three parameters were deemed to be deficient in BRCA1 function.

The exclusion of promoter methylation analysis from this study was due to the fact that in the cases with hypermethylation (almost exclusively TNBCs) the BRCA1:ID4 ratio and protein presence and localization were able to classify the tumor into the appropriate BRCA1 category. We were also concerned that the use of MethyLight would add a complex assay to the mix making its broader adaptation more challenging.

While we acknowledge that the ideal test would be one that demonstrates functional deficiency of HR mediated DSB repair in fresh clinical specimens, we have worked towards a practical strategy that would permit the identification of tumors with low or absent BRCA1 transcript and protein.

The present identification of BRCA1 deficiency based on the knowledge of BRCA1 regulation will have to await confirmation in an independent cohort, followed by clinical validation before being adopted widely. In addition, though the absolute numbers of TNBCs was rather limited, the proportion of sample being categorized as deficient in BRCA1 was 40% and corresponded to the proportion of high ID4 immunoreactivity observed in the MSKCC study that examined twice the number of TNBCs we have used in this study.

## Supporting Information

S1 Fig Histograms depicting distributions of transcript levels of—A. BRCA1 and B. ID4. (DOCX)

S2 Fig Images- Immunohistochemistry. (DOCX)

S3 Fig Correlation of MIR182 to BRCA1 protein. (DOCX)

S4 Fig KaplanMeir Curves for survival analysis. (DOCX)

S1 Table Comparison of clinical characteristics of the BRCA1 deficient and adequate groups in the 55 TNBC samples. (DOCX)

## Abbreviations

TNBC: Triple Negative Breast Cancer
BLBC: Basal like breast cancer
HR+: Hormone receptor positive
IHC: Immunohistochemistry
aRNU: Adjusted Relative normalized units
TCGA: The Cancer Genome Atlas
