# Supplementary material for: Identification of BRCA1 Deficiency Using Multi-Analyte Estimation of BRCA1 and Its Repressors in FFPE Tumor Samples from Patients with Triple Negative Breast Cancer
Source: PLoS One. 2016 Apr 14;11(4):e0153113. doi: 10.1371/journal.pone.0153113 (PMC4831669; doi:10.1371/journal.pone.0153113)
Supplement: S1 Fig — (DOCX) [file pone.0153113.s001.docx]

**S1 Figure**: **Histograms depicting distributions of transcript levels of - A. BRCA1 and B. ID4**


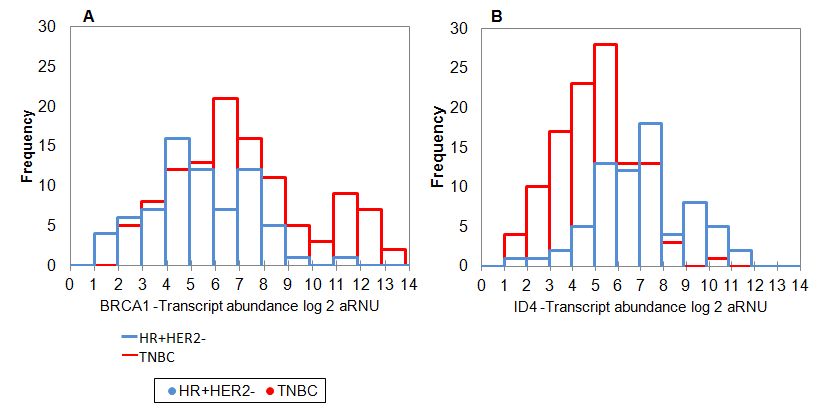


Distribution of Transcript levels of BRCA1 and ID4 in the two groups of HR+ HER2- and of TNBC. Total number of samples: n=183; HR+HER2- = 112, TNBC=71. A right shift of high BRCA1 observed in the HR+HER2- class and right shift of high ID4 in the TNBC class exhibiting a reciprocal relationship. (OR- The high ranges of BRCA1 are exclusively HR+, and the high-ranges of ID4 are all TNBC)
