# Supplementary material for: Identification of BRCA1 Deficiency Using Multi-Analyte Estimation of BRCA1 and Its Repressors in FFPE Tumor Samples from Patients with Triple Negative Breast Cancer
Source: PLoS One. 2016 Apr 14;11(4):e0153113. doi: 10.1371/journal.pone.0153113 (PMC4831669; doi:10.1371/journal.pone.0153113)
Supplement: S2 Fig — (DOCX) [file pone.0153113.s002.docx]

**S2 Figure: Images- Immunohistochemistry**


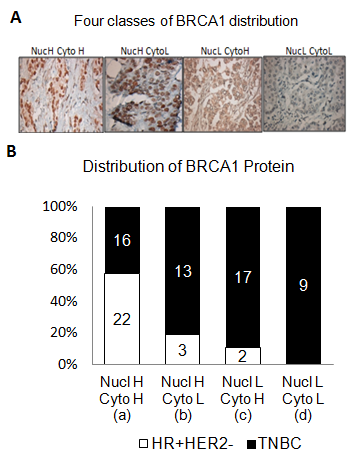


**C**


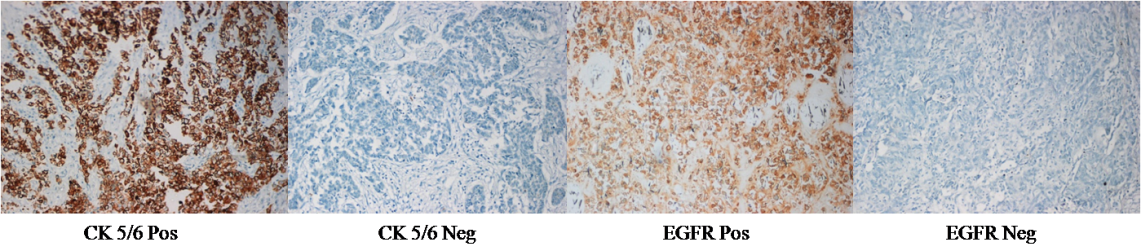


BRCA1 Protein regulation. **A**. IHC images of distribution of BRCA1 protein in the four classes. **B**. Percentage distribution of BRCA1 protein into four different patterns of localization- Nuclear (Nuc) and Cytoplasmic (Cyto) either high (H) or low (L) in HR+ and TNBC. The initial 4 category classification was collapsed into a two category based on only the nuclear presence and disregarding the cytoplasmic levels as suggested by Jorge R Filho, Pathologist, MSKCC (personal communication). We clubbed the two nuclear high groups (a & b) into the BRCA1 protein adequate group, and the two nuclear low groups (c &d) into the BRCA1 deficient protein group. **C.** Immunohistochemistry of CK5/6 and EGFR.
