# Supplementary material for: Identification of BRCA1 Deficiency Using Multi-Analyte Estimation of BRCA1 and Its Repressors in FFPE Tumor Samples from Patients with Triple Negative Breast Cancer
Source: PLoS One. 2016 Apr 14;11(4):e0153113. doi: 10.1371/journal.pone.0153113 (PMC4831669; doi:10.1371/journal.pone.0153113)
Supplement: S3 Fig — (DOCX) [file pone.0153113.s003.docx]

**S3 Figure: Correlation of MIR182 to BRCA1 protein**

**
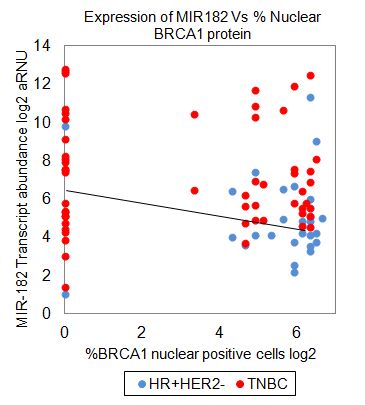
**

**S3 Figure:** The percentage of BRCA1 nuclear positive cells was transformed to a log2 scale with values between 0-6.7. All tumors scored negative (<10) were given a value of 0. The MIR182 transcript was plotted against the BRCA1 protein values. An inverse relationship was observed between transcript levels of MIR182 and BRCA1 Protein. n=82
