# Supplementary material for: Identification of BRCA1 Deficiency Using Multi-Analyte Estimation of BRCA1 and Its Repressors in FFPE Tumor Samples from Patients with Triple Negative Breast Cancer
Source: PLoS One. 2016 Apr 14;11(4):e0153113. doi: 10.1371/journal.pone.0153113 (PMC4831669; doi:10.1371/journal.pone.0153113)
Supplement: S4 Fig — (DOCX) [file pone.0153113.s004.docx]

**S4 Figure : KM Curves for survival analysis**

**
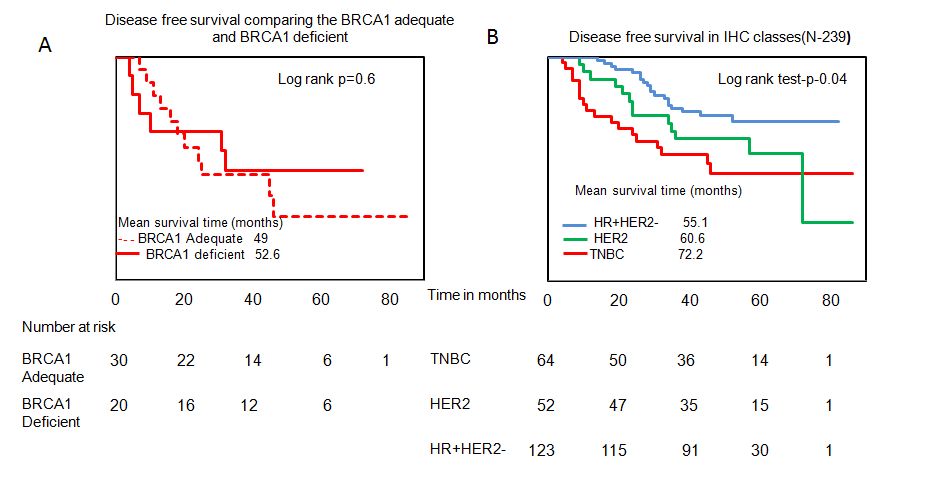
**

**S4 Figure : A**: Kaplan Meir curves for survival analysis in the TNBC group between the BRCA1 deficient and BRCA1 Adequate groups. BRCA1 adequate n=30, and BRCA1 deficient group, n=20. The cumulative events at 60 months are (n=17) 34%. 4/17 patients who have had events were not treated by standard of care regimens. None of the patients were treated with Platinum. **B**: KM curves for survival analysis in the entire case series separated by the three IHC classes. The cumulative disease related events so far are (n=50) = 21%. The subgroups behave exactly as was expected of them with HR+HER2- having the best survival and TNBCs the least.
