# Supplementary material for: Identification of BRCA1 Deficiency Using Multi-Analyte Estimation of BRCA1 and Its Repressors in FFPE Tumor Samples from Patients with Triple Negative Breast Cancer
Source: PLoS One. 2016 Apr 14;11(4):e0153113. doi: 10.1371/journal.pone.0153113 (PMC4831669; doi:10.1371/journal.pone.0153113)
Supplement: S1 Table — (DOCX) [file pone.0153113.s005.docx]

**S1 Table : Comparison of clinical characteristics of the BRCA1 deficient and adequate groups in the 55 TNBC samples**

|  | Deficient  N=22 (%) | Adequate N=33(%) |
| --- | --- | --- |
| Median Age | 49 | 54 |
| Median Tumor size | 3.3 | 3.5 |
| LN Positive | 11 (51) | 18 (54) |
| LN Negative | 10 (45) | 14 (42) |
| Unknown | 1 | 1 |
| Grade I | 0 (0) | 1 (3) |
| Grade II | 6 (28) | 13 (39) |
| Grade III | 14 (63) | 18 (55) |
| Unknown | 2 | 1 |
| Basal By IHC | 15 (68) | 17 (51) |
